# Current Trends Featuring the Bridge Between Stroke and End-Stage Renal Disease: A Review

**DOI:** 10.7759/cureus.9484

**Published:** 2020-07-30

**Authors:** Radhika Akku, Thulasi Priya Jayaprakash, Olisaemeka D Ogbue, Parul Malhotra, Safeera Khan

**Affiliations:** 1 Internal Medicine, California Institute of Behavioral Neurosciences & Psychology, Fairfield, USA; 2 Medicine, California Institute of Behavioral Neurosciences & Psychology, Fairfield, USA; 3 Internal Medicine, Punjab Institute of Medical Sciences, Ludhiana, IND

**Keywords:** end-stage renal disease (esrd), stroke complications

## Abstract

The relationship between end-stage renal disease (ESRD) and cerebral stroke is graded and cumulative, having a significant impact on morbidity and mortality. Ischemic stroke is more prevalent than hemorrhagic stroke and both stroke types have modifiable and non-modifiable risk factors. The presence of risk factors such as hypertension, diabetes, and atrial fibrillation (AF) before stroke occurrence in dialysis patients has a significant impact on the outcomes such as a discharge to rehabilitation, in-hospital mortality with the worst prognosis when compared to the general population. ESRD patients with either peritoneal or hemodialysis (HD) are at increased risk of stroke than the general population, with a high mortality rate at the commencement of dialysis and gradually decreases. Primary and secondary prevention of risk factors plays a significant role in this susceptible population and helps to mitigate better treatment and outcomes. Our review article focuses on the mechanisms, outcomes, treatment, and preventive aspects of stroke in the ESRD population.

## Introduction and background

Stroke and end-stage renal disease (ESRD) are a global health burden, and the incidence of stroke in ESRD has been rapidly increasing over the years [1]. There is not only a higher risk of death in stroke patients but also significant years of life lost [2]. Cardiovascular disease (CVD) is one of the major causes of morbidity and mortality in dialysis patients [3]. The risk of stroke in the ESRD population is about 30-fold compared to the general population and is the third most common cause of death. The risk of hospitalization from ischemic or hemorrhagic stroke is 4-10 times greater with poor long-term prognosis. Increased risk of death from stroke in ESRD patients is seen mainly in women and young people with dialysis initiated early during treatment [4-6]. Studies have shown that in patients undergoing dialysis, stroke prevalence is high at the initiation of dialysis with high incidence in the follow-up. A stroke occurs in approximately 70% of the ESRD population [3,7]. The risk of ischemic stroke in the hemodialysis (HD) group of patients is significantly higher than that seen in peritoneal dialysis (PD) group. Still, no significant difference in the risk of hemorrhagic stroke was observed in both groups [1]. Deaths in the ESRD group are higher in patients with hemorrhagic stroke than ischemic stroke [4].

The literature infers, increased atherosclerosis of large blood vessels, and small vessel injury is one of the factors contributing to the specific stroke features in the ESRD population [8]. Chronic kidney failure (CKD) by itself increases stroke risk apart from the known risk factors [9]. Modifiable risk factors include hypertension, diabetes, CVD, obesity, smoking, and alcohol. Non-modifiable risk factors include the elderly population, male gender, family history, non-white race, and non-caucasian ethnicity [6,10,11]. Effective control of atherosclerotic risk factors such as hypertension, diabetes, cholesterol levels, and calcium-phosphorus metabolism may be helpful for stroke prevention in patients undergoing dialysis [1]. The risk of stroke increases proportionately with an increase in both systolic and diastolic blood pressure (BP), and thus, hypertension remains the most modifiable risk factor [6,12]. To improve clinical outcomes in ESRD patients, emphasis should be on primary and secondary prevention of cardiovascular risk factors as stroke is associated with adverse outcomes in this high-risk group [13].

In high-income countries, the stroke rate is reduced compared to low- to middle-income countries. The highest risk for initial stroke is during the initiation of dialysis. Factors increasing the risk of stroke constitute the vascular comorbidities resulting from renal failure and factors associated with renal failures like modes of dialysis, vascular access, anticoagulation use for extracorporeal circuit flow maintenance, the impact from uremic toxins, and malnutrition-inflammation-atherosclerosis complex [14]. Some of the other factors contributing later for stroke occurrence in ESRD are inflammation, oxidative stress, anemia, reduced nitric oxide, aggregation of platelets, and high homocysteine levels, causing endothelial dysfunction [9]. Hemorrhagic stroke patients on dialysis should be monitored very closely as they are more prone to bleeding due to uremia and heparin use [15]. Increased bleeding risk causes a significant burden not only on the patient but on family and society as well [2].

Thus, stroke prevention in ESRD is essential to decrease the duration of hospital stay and associated mortality and other effects such as vascular cognitive impairment or dementia [16]. Not much is known about stroke prevention and how stroke affects mortality in ESRD patients. Our study focuses on determining the effects of stroke on the mortality in patients with ESRD. 

## Review

Methods

Literature Search

We performed a literature search on PubMed using medical subject heading (MeSH) terms to identify relevant studies. Studies that were published in the last ten years, humans only, adults 19+, and in the English language were included. The MeSH terms including “end-stage renal disease,” “stroke complications, prevention, and control” were used. The Boolean operators “AND” was used to facilitate the search. The reference lists of retrieved articles were manually searched to identify related articles. The latest date of the search was June 27, 2020, without using Preferred Reporting Items for Systematic Reviews and Meta-Analyses (PRISMA) guidelines.

Inclusion Criteria

The following inclusion criteria were used: (a) this is a review article comparing the effects of stroke on mortality in ESRD patients with PD vs. HD; (b) the outcomes of interest were post-stroke events.

Exclusion Criteria

The following exclusion criteria were used: (a) the inclusion criteria were not met; (b) the study population included pediatric patients; and (c) the studies were duplicate articles, commentaries, editorials, case reports, and case series. 

Results

Nine studies that investigated stroke risk and remaining studies that investigated mechanisms, outcomes, and management were all reviewed in detail. Two studies were systematic reviews and meta-analysis comparing stroke risk in patients with PD and HD [17,18]. A study conducted by Zhan et al. compared stroke risk in the Asian and non-Asian populations and showed no difference in risk [17]. A study conducted by Boonpheng et al. showed that PD patients had low hemorrhagic stroke risk and no change in ischemic stroke compared to HD patients [18]. There are five retrospective cohort studies. One study showed increased stroke risk compared to the general population [19]. A study conducted by Wetmore et al. showed increased hemorrhagic stroke risk in African Americans [20]. The incidence of hemorrhagic stroke was more than ischemic stroke in South Asian ethnicity [21]. Chen et al. performed a retrospective cohort study that showed low hemorrhagic risk in PD with high lipoprotein [22]. One prospective cohort study evaluated the risk of hemorrhagic transformation in ischemic stroke [23]. A retrospective study showed hemorrhagic or ischemic stroke seen more in the elderly population [24]. Whereas a prospective study showed an increased risk of HT with the progression of renal dysfunction [23,25]. Table 1 presents the risk of stroke in dialysis patients in relevant studies [17-25].

**Table 1:** Risk of stroke in dialysis patients in relevant studies. GFR: Glomerular filtration rate, HT: hemorrhagic transformation, PD: peritoneal dialysis, HD: hemodialysis, OR: odds ratio, CI: confidence interval, HR: hazard ratio, ARR: absolute risk reduction, ESRD: end-stage renal disease; LP: lipoprotein, TT: thrombolytic therapy, AF: atrial fibrillation, RR: rate ratio.

Author name	Year of publication	Type of study	Number of patients	Purpose of the study	Results/conclusions
Zhan et al [17]	2019	Systematic review and meta-analysis	1,219,245	Comparing stroke risk in patients with PD and HD	For non-Asian patients, PD was associated with low risk of hemorrhagic stroke (HR ¼ 0.78; 95% CI: 0.69–0.88; p < 0.001) and high risk of ischemic stroke (HR ¼ 1.13; 95% CI: 1.05–1.23; p ¼ 0.002) compared to HD. In Asian patients, no statistically significant difference is seen. For overall stroke risk, there was no difference between PD and HD.
Boonpheng et al. [18]	2018	Systematic review and meta-analysis	1,289,572	Comparing stroke risk in patients with PD and HD	PD patients showed 16% low risk of hemorrhagic stroke with pooled OR of 0.84 (95% CI: 0.76-0.92) and no statistically significant difference with all types of stroke or ischemic stroke with pooled ORs of 1.06 (95% CI: 0.91-1.22) and 1.01 (95% CI: 0.80-1.18), respectively, when compared to HD patients.
Wang et al. [19]	2014	Retrospective cohort study	7,49,939	Risk of stroke in dialysis patients compared to the general population	Incidence of in-hospital ischemic stroke (102.6 and 100.1/10,000 person-years) and hemorrhagic stroke (74.7 and 59.4/10,000 person-years) was high in PD and HD patients after adjusting age and sex-matched cohort (42.4 and 13.0/10,000 person-years). In both types of stroke hypertension, diabetes, male sex, and old age are independent risk factors and thus control of diabetes and hypertension is important in dialysis patients.
Wetmore et al. [20]	2014	Retrospective cohort study	265,685	Comparing hemorrhagic stroke in dialysis patients	2,397 (0.9%) patients had a hemorrhagic stroke over a median follow-up of 15.5 months. Compared to whites, African Americans (ARR, 1.43; 95% CI: 1.30-1.57), Hispanics (ARR, 1.78; 95% CI: 1.57-2.03), and individuals of other races (ARR, 1.51; 95% CI: 1.26-1.80) had a significantly higher risk for hemorrhagic stroke. Thus, ethnicity and race contribute to a significant difference in hemorrhagic stroke in dialysis patients.
Power et al [21]	2012	Retrospective cohort study	2,384	Comparing ischemic stroke with hemorrhagic stroke in dialysis patients	The ischemic stroke occurred at a higher rate compared to hemorrhagic stroke (11.2 vs. 3.7/1,000 patient-years). In South Asian ethnicity, 54% of patients had hemorrhagic strokes with predominantly seen in white patients (45% of events). Incident cerebrovascular accident were seen more in patients with diabetes mellitus (HR, 1.92; 95% CI: 1.29-2.85; P = 0.001) and prior cerebrovascular disease (HR, 4.54; 95% CI: 3.07-6.72; P < 0.001). One-year mortality was 24% overall in patients with acute stroke and poor survival (HR, 3.26; 95% CI: 2.47-4.30; P < 0.001). Hemorrhagic events were 39% vs. 19% mortality for ischemic events.
Chen et al. [22]	2019	Retrospective cohort study	860	Risk of hemorrhagic stroke in patients with PD	In patients with incident PD, high-Lp(a) group had a significantly low risk of hemorrhagic stroke compared with low Lp(a).
Lee et al. [23]	2013	Prospective cohort study	770	Increased risk of HT in ischemic stroke patients with low GFR	Decreased GFR increases symptomatic HTs and significant association between GFR <30 groups and HTs in acute ischemic stroke (OR 2.90; 95% CI: 1.26-6.68, p = 0.012) after adjusting for other risk factors was seen
Ozelsancak et al. [24]	2019	Retrospective observational study	432	Risk factors for stroke in HD patients	Ischemic and hemorrhagic lesions were seen in 65 (24.62%) and 25 (9.47%) patients, respectively. Diabetes was the cause in 58.5% of patients with ischemic lesions and 52% of patients with hemorrhagic lesions leading to ESRD. Stroke in HD patients is usually seen in older age groups with low serum creatinine and diabetes.
Liu et al. [25]	2018	Prospective study	426	Low GFR is an independent predictor of HT in acute ischemic stroke patients without TT	Apart from low GFR, additional risk factors increasing HT include large infarct volume, AF, and hypoalbuminemia.

Discussion

Mechanisms of Stroke in ESRD Patients

The interrelation of the kidney and brain involves central and peripheral vascular systems and has anatomical and physiological factors making them susceptible to cardiovascular risk factors [16,26]. CKD itself increases stroke risk. Thus, it is important to understand the effect of stroke in ESRD patients [27]. Masson et al. performed a systematic review and meta-analysis of 83 studies that showed stroke risk increases by 7% for every 10 mL/min/1.73 m^2^ decrease in glomerular filtration rate (GFR). This suggests that there is a linear relation between declining GFR and stroke risk [28]. Mechanisms involved in stroke pathogenesis in ESRD include systemic inflammation, arterial stiffness, risk of AF, platelet and endothelial dysfunction, homocysteinemia, accelerated atherosclerosis, and uremia [29]. Thrombus formation in ESRD is mediated by endothelial damage as the endothelium balances procoagulant and anticoagulant pathways. Endothelium prevents the occurrence of thrombi by the formation of nitric oxide, prostacyclins, and tissue plasminogen activator (tPA). Patients with ESRD have chronic inflammation through the release of inflammatory markers such as interleukin-6, C-reactive protein, and fibrinogen. Thus, there is a linear relationship between the progression of kidney disease and endothelial dysfunction. Platelet binding to fibrinogen and Von Willebrand factor is decreased from reduced glycoprotein IIb-IIIa receptors, and there is an increased circulation of thrombotic factors [30]. 

**Figure 1:**
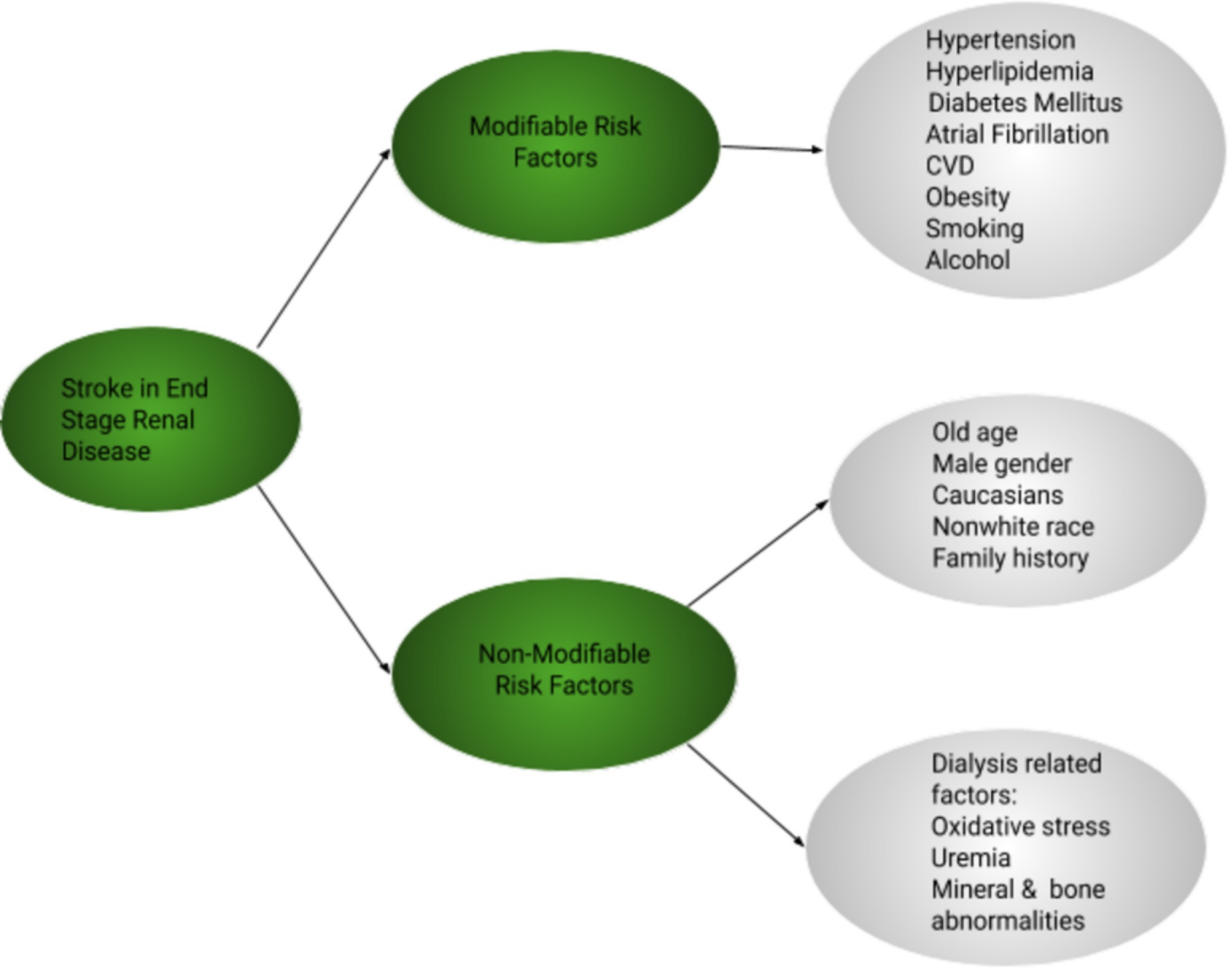
Risk factors for stroke in ESRD patients. CVD: cardiovascular disease, ESRD: end-stage renal disease.

Uremia in ESRD patients not only causes platelet dysfunction but also the toxins cross the blood-brain barrier and are involved in stroke pathogenesis [26]. There is an increased risk of atherosclerosis in ESRD from a combination of factors that contribute to stroke. Studies have shown that the occurrence of atherosclerosis of carotid arteries in dialysis patients is more compared to the general population. Homocystenemia further adds to stroke risk by causing endothelial damage, atherosclerosis, and thrombus formation [29]. Thus, preventing endothelial damage may benefit ESRD patients in preventing stroke occurrence. Figure 1 demonstrates modifiable and non-modifiable risk factors of stroke in ESRD.

**Figure 2:**
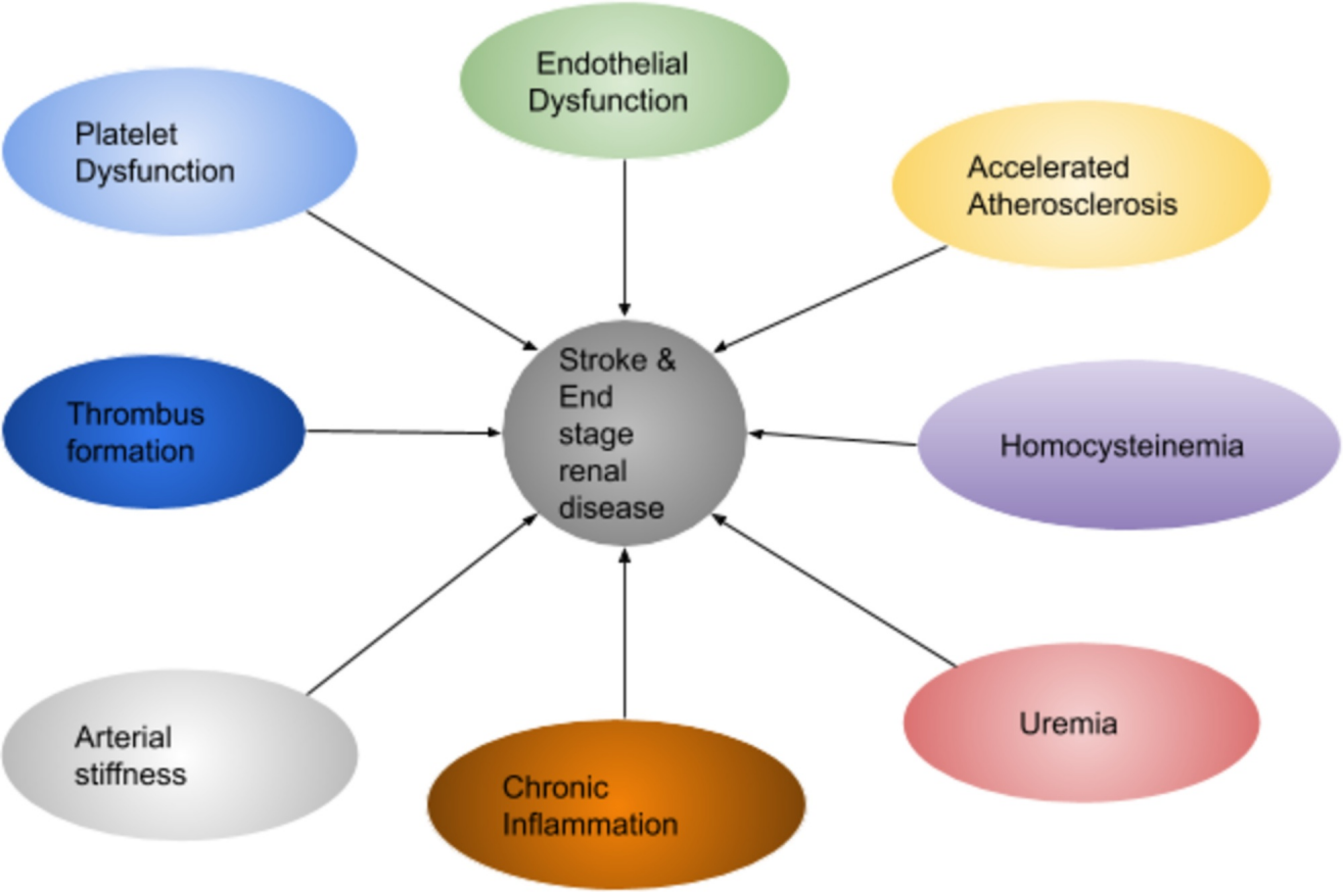
Pathogenesis of stroke and ESRD. ESRD: end-stage renal disease.

Figure 2 shows the factors involved in the pathogenesis of stroke in ESRD.

Mortality and Outcome of Stroke in ESRD

One of the most significant factors responsible for the association between stroke and ESRD is mediated by aggregation of traditional and non-traditional risk factors [6]. Cohort study with 770 patients done by Lee et al. showed that the risk of HT (OR, 2.90, 95% CI: 1.26-6.68, p = 0.01) after an ischemic stroke was associated with advanced CKD (eGFR <30 ml/min) [23]. This suggests that CKD serves as an independent risk factor for hemorrhagic stroke. A study conducted by Ovbiagele showed an increased risk of in-hospital mortality (OR, 1.63, 95% CI: 1.52-1.75, p < 0.001) in CKD patients and the association is more pronounced with ESRD [31]. There is a threefold higher risk of death in HD patients after an acute ischemic stroke [21,31].

Mata et al. conducted a cohort study with 60,823 patients, and follow-up showed 941 stroke deaths and 33,377 non-stroke deaths. Risk of death from stroke was higher with old age (SHR, 1.92; 95% CI: 1.45-2.55), in females (SHR, 1.41; 95% CI: 1.21-1.64), cerebrovascular disease (SHR, 2.39; 95% CI: 1.99-2.87), ESRD due to hypertensive/renovascular disease (SHR, 1.39; 95% CI: 1.09-1.78), polycystic kidney disease (SHR, 1.38; 95% CI: 1.00-1.90), and with an earlier year of dialysis initiation (SHR 1.93; 95% CI: 1.56-2.39). These patients can benefit from preventive management as they are a high-risk group [4].

Increased risk of all-cause mortality is seen in ESRD patients already diagnosed with peripheral artery disease. Deaths from stroke and non-stroke causes are enhanced in the old age group, early dialysis initiation, normal body mass index (BMI) than obesity, prior cerebrovascular disease, HD than transplant patients, diabetes, or kidney failure caused by hypertension/renal artery disease than glomerulonephritis/IgA nephropathy. Further analysis showed that for stroke-related deaths, females are at increased risk and for non-stroke deaths low BMI, white race, smoking, coronary artery disease, and renal failure due to glomerulonephritis/IgA nephropathy are risk factors [5]. The risk of stroke mortality is more in women and younger age groups [4]. A study conducted by Ovbiagele on one million stroke hospitalization showed that patients with CKD (6.1%) had increased in-hospital mortality (9%) with either stroke types [31]. This effect was particularly seen in females and young age groups [31]. 

Compared to the general population, HD patients have eight to ten times increased incidence of stroke [6]. Studies have reported that stroke is one of the leading causes of mortality in dialysis patients. A cohort study conducted by Cohen-Hagai et al. showed that ischemic strokes are common in the HD group compared to the general population with the unfortunate outcome due to longer duration of hospital stay, decreased nutritional condition at discharge, no change in recovery despite rehabilitation programs, and higher mortality [32]. HD patient groups had the presence of comorbidities such as hypertension, diabetes, congestive heart failure, left ventricular hypertrophy (LVH), AF, peripheral vascular disease, and pulmonary disease more than the general population. The OR for death in HD group of stroke patients compared to the general population with stroke was 2.53 (CI: 1.4-4.58) with increased mortality at the hospitalization phase, at 30 days and one year after the stroke [32]. 

Among the ESRD patients, the most vulnerable population is among the developing countries due to the expenses involved in patient care and renal replacement therapy. One study showed that there is a significantly increased risk of stroke incidence among Asians than the non-Asian population. In the ESRD population, there are limitations to treatment after either ischemic or hemorrhagic stroke that include the use of oral anticoagulants, antithrombotics, reperfusion therapy after ischemic stroke. This suggests that ESRD is one of the significant factors contributing to neurological outcomes [27].

Most patients undergoing dialysis are older with the presence of either cardiovascular risk factors or coexisting CVD, which makes them prone to either hemorrhagic or ischemic stroke. Ischemic stroke constitutes about 70% of stroke subtype and has a poor short- and long-term prognosis in dialysis patients. Thus, increasing renal dysfunction increases ischemic stroke risk. Rao et al. conducted a cohort study on 18,320 ischemic stroke patients treated with tPA that showed increased odds of in-hospital mortality in patient switch eGFR <45 (6.7% vs. 0.9%, adjusted OR, 3.59; 95% CI: 2.18-5.91), eGFR 45-59 (4.0% vs. 0.9%, adjusted OR, 2.00; 95% CI: 1.18-3.38) and no statistically significant difference for symptomatic intracerebral hemorrhage with eGFR <45 (OR, 0.86; 95% CI: 0.59-1.24) and eGFR 45-59 (OR, 1.08; 95% CI: 0.79-1.48), respectively [33]. This suggests that in-hospital mortality increases, especially with eGFR <45 in patients with ischemic stroke and no association with an increase in-hospital symptomatic intracranial hemorrhage and eGFR was seen. Therefore, tPA therapy can be used in CKD patients [33].

Ischemic stroke is more prevalent at the time of dialysis initiation, and the incidence during the follow-up period is increased. Sanchez-Perales et al. reported in a study that included 449 patients, 30 had a prior stroke before starting dialysis, and 34 had one or more strokes. AF is an independent predictor of stroke occurrence (OR: 3.11; 95% CI: 1.53-6.32; P = 0.002). This study showed that one of the major predictors of ischemic stroke is AF, and anticoagulation therapy for stroke prevention must be given after analyzing risks and benefits. The predominant causes of death in dialysis patients are from cardiovascular diseases, with most occurring from ischemic heart disease and LVH. In this study, AF was seen in a quarter of dialysis patients with prior stroke, and 20 of 34 stroke patients in follow-up had AF before starting dialysis or during the follow-up. Thus, AF contributes to an important risk factor for ischemic stroke in dialysis patients [7].

Cherng et al. conducted two nationwide studies on 1,378 ESRD patients for stroke incidence and 318,638 hospitalized stroke patients for post-stroke mortality. The results showed that the hazard ratio (HR) after propensity-score matching for stroke incidence in ESRD patients was 2.08 (95% CI: 1.32-3.26), and adjusted rate ratio (RR) for post-stroke mortality was 2.62 (95% CI: 2.43-2.82) compared to control. Stroke risk was increased in patients with diabetes, hypertension, and post-stroke mortality was seen more in patients with comorbidities anemia, diabetes, ischemic heart disease, or liver cirrhosis compared to controls. Due to high statin use and pharmacotherapy, hyperlipidemia, and ischemic heart disease, heart failure did not show much effect on stroke or stroke mortality. Thus, ESRD is an independent risk factor for stroke [34]. 

Alqahtani et al. conducted a cohort study on 9,30,010 patients which showed ischemic stroke incidence in patients on maintenance dialysis has decreased over the years by 36% compared to non-dialysis patients; the incidence has unchanged. Dialysis patients included more percentage of young age with the presence of comorbidities, females, African-Americans, and Hispanics. In-hospital mortality was high but decreased over the years; blood transfusion, sepsis, and length of hospital stay were higher with 25% more cost for care; hemorrhagic conversion, and rates of gastrointestinal bleeding were the same in the dialysis group. This suggests that in the dialysis group, more preventive therapies are required to avoid complications [35]. 

Wu et al. compared the risk of developing CKD and ESRD in stroke and without stroke patients showed that the presence of comorbidities diabetes mellitus, hyperlipidemia, and gout (aHRs of 2.12 vs. 1.31, 1.53 vs. 1.47 or 1.84 vs. 1.46) increased the risk of progression to ESRD. Stroke serves as an independent predictor for the progression of CKD to ESRD [11]. Compared to the elderly population, CKD was seen more in stroke patients <50 years. Risk factors causing CKD and progression to ESRD are greater in ischemic stroke patients (aHRs of 1.66 vs. 1.41) than hemorrhagic stroke. Thus, these patients need to be monitored closely as stroke occurrence at an early age might lead to prolonged exposure to risk factors and adds to a decline in kidney function as stroke and CKD have the same pathophysiology [36]. Atherosclerosis and vascular factors affect renal and cerebral function by ischemia, disruption of the endothelium, and blood-brain barrier [27]. Studies have shown that stroke risk increases with CKD progression regardless of the vascular comorbidities. Patients with CKD and ESRD have limitations with stroke treatment due to decreased medicine clearance, greater predisposition to bleeding, and thus more adverse effects from management [29]. 

Wang et al. conducted a cohort study that showed a low risk of hemorrhagic stroke (HR, 0.75; 95% CI: 0.58-0.96) and the same risk of ischemic stroke in patients on PD compared to HD [19]. A cohort study on 69,371 dialysis patients showed more ischemic strokes than hemorrhagic, and mortality for hemorrhagic stroke during the 30 days follow-up was greater than ischemic stroke and mortality decreased over the years, and no difference in race was seen. Factors causing burden and contributing to morbidity in long-term survivors of stroke patients include factors such as myocardial infarction and hip fracture [2]. 

Tollitt et al. did a cohort study in 3,060 patients which showed an association between prior stroke and mortality, a progression of CKD to ESRD, and the occurrence of non-fatal cardiovascular events. Patients with stroke at the start of dialysis had poor outcomes, higher all-cause mortality, and decreased survival than patients not on dialysis. This signifies the important association between cerebral and renal pathways to evaluate the outcomes in dialysis patients [13]. Ovbiagele et al. conducted a cohort study on 6,79,827 hospitalized ischemic stroke patients. As there is a progression of renal dysfunction to ESRD, there is a decline in the rate of the following treatment protocol, receiving adequate therapy, and there was more significant in-hospital mortality [36].

A study conducted by Findlay et al. on 4,957 dialysis patients showed that stroke incidence was seen in females, diabetes, the elderly age group, hypertension, and HD showed increased association with stroke risk compared to PD and served as an added risk factor. Stroke risk is about ten times greater in patients undergoing renal replacement therapy compared to the general population [37]. 

Some of the mechanisms suggested for the occurrence of a vascular event in a susceptible patient undergoing transplantation include fluid shifts, BP changes, use of immunosuppressants, and anesthesia, which might play a role of a stressor [37]. Increased risk of complications such as the HT of ischemic stroke, prolong hospital stay, in-hospital mortality, and infections can occur with a decline in GFR and progression to ESRD.

Management

Stroke prevalence is about 38%, and stroke in patients with renal dysfunction is mostly mediated by atherosclerosis. ESRD contributes to a significant health burden all over the world. Stroke is the most common neurological disorder occurring in ESRD patients with an ischemic stroke occurring in 70% of patients. The presence of comorbidities has shown a significant association with the progression of kidney dysfunction and has short- and long-term mortality [3]. Studies have shown conflicting evidence regarding the clinical outcome of thrombolytic treatment in CKD patients with acute ischemic stroke. One of the main complications is hemorrhagic infarction with thrombolytics apart from HT and cerebral microbleeds. Treatment is individualized based on patient comorbidities, and the potent treatment for stroke is still limited [8].

Stroke prevalence was about eight times more than the general population, and the prevalence at the commencement of dialysis was 6.7%. One of the most important factors causing ischemic stroke in ESRD is AF, and most of these patients are old with coexisting comorbidity [34]. 

A cohort study conducted by Cohen-Hagai et al. in HD patients reported 52 strokes (45 ischemic and seven hemorrhagic). All patients had the presence of comorbidities congestive heart failure (CHF), hypertension, diabetes, chronic AF, chronic lung disease, peripheral vascular disease, and LVH and received treatment with tPA, aspirin, alpha, and beta-blockers. Stroke occurred mostly within a month of starting dialysis, and the results can be biased as it only involved caucasian race patients. This study showed that stroke prevalence was greater than ten times higher than the general population. Patients used antihypertensive medications due to the high prevalence of AF and LVH. Patients in the HD group with AF had a high CHAD VAS score, with 4.1% receiving anticoagulants (besides heparin during dialysis), and 67.3% were on low dose aspirin and antiplatelet therapy. tPA was given only to four HD patients with stroke occurrence in the hospital after neurological workup compared to stroke patients with normal renal function. Patients who received tPA had no neurological complications. At the functional discharge status of HD, patients were poor compared to normal renal function. Thus, most HD patients were discharged for home rehabilitation compared to hospital rehabilitation in normal renal function due to decreased likelihood of recovery. OR for death was 2.53 (CI: 1.4-4.58) in HD stroke patients compared to stroke in patients with normal renal function. Stroke is the most important factor contributing to mortality in HD patients [32].

There were 2.5 times greater odds of in-hospital mortality in patients with eGFR < 15 with no dialysis compared to eGFR ≥ 60. Dialysis patients had a better stroke outcome compared to non-dialysis patients suggesting any possibly severe associated comorbidities or poor health conditions from the beginning that can prevent them from dialysis. There is a decrease in-hospital mortality rate from 45% to 20% in women compared to men from eGFR < 15 to eGFR 15-29 [38]. CKD serves as an independent risk factor for ischemic and hemorrhagic stroke despite many other risk factors [8]. Thus, the management of these patients is important. In CKD patients with AF, warfarin anticoagulation with INR target 2.0 and 3.0 for embolic stroke prevention and avoiding major bleeding can be useful [8].

Even though there is an increased risk of stroke in patients with CKD, there are no specific guidelines for antiplatelet treatment. CKD patients are prone to the risk of bleeding and inadequate response to antiplatelets; aspirin can aggravate kidney dysfunction; thus, treatment must be individualized. Other aspects of treatment regimen are control of BP, lipid-lowering agents, and renal transplantation. Since endothelial dysfunction is one of the mechanisms involved in stroke pathogenesis in CKD, therapy to reduce endothelial dysfunction such as statins, angiotensin-converting enzyme (ACE) inhibitors, phosphodiesterase inhibitors (such as cilostazol and dipyridamole) to enhance nitric oxide release can be helpful. In diabetes, to slow down the progression of diabetic nephropathy ACE inhibitors and angiotensin II receptor antagonists are first-line agents. The most effective treatment is renal transplantation, and side effects with immunosuppressants after transplantation must also be taken into account [9]. The target BP in HD patients before dialysis is 140/90 mmHg [12]. After an acute stroke, the initial target is to preserve function at-risk cerebral areas and avoid further injury to prevent complications such as seizures, increased intracranial pressure, hyponatremia, and coagulopathy from anticoagulants [39]. In ESRD patients with AF, warfarin anticoagulation with INR target 2.0 and 3.0 for embolic stroke prevention and avoiding major bleeding can be useful. Compliance with statins for hyperlipidemia, hypertension, and diabetes control in this vulnerable population may help in stroke occurrence. 

Prevention

Control of hypertension, diabetes, triglycerides, CVD, calcium-phosphorus metabolism is some aspects of stroke prevention [1]. In AF patients receiving warfarin, there is an increased risk of intracerebral vascular calcification [6,14]. Even though TT can be used in all stages of CKD, its beneficial effect is decreased with the progression of kidney disease. Hemorrhagic stroke is caused by small vessel rupture mediated by hypertension and ischemic stroke atherosclerosis with thrombus formation and embolization [40]. BP control and antiplatelets are important aspects of the prevention of stroke. The risk of bleeding in dialysis patients should be weighed against the benefit of anticoagulants and antiplatelet use. There is no ideal BP for stroke prevention. BP management is the mainstay for primary and secondary prevention of stroke not only in ESRD but also in non-dialysis patients. In patients with carotid stenosis, especially high-grade (>70%), carotid endarterectomy is favored to decrease the risk of stroke. 

Limitations

A few limitations of our study were that we only included humans studies, studies published in the last ten years, only in the English language and included cohort studies, case-control studies, review articles, systematic review, and meta-analysis. Thus, our study might have missed some of the important studies on the effect of stroke in ESRD patients.

## Conclusions

In conclusion, outcomes of strokes are compelling in ESRD patients with either HD or PD, causing a substantial effect. Due to the higher frequency of stroke in patients with CKD and dialysis than the general population, dialysis patients should be monitored from the initial stages. Even though ischemic stroke is more common than hemorrhagic stroke, HT in ischemic stroke patients has a poor outcome, and thus, they should be monitored closely. Despite HD and PD having different pathophysiology involved, compliance with medications such as warfarin therapy in AF, ACE inhibitors, statins in patients with myocardial infarction, congestive heart failure, diabetes and hypertension control, and clopidogrel is required for better survival. Focus on the treatment of established risk factors in dialysis patients for the prevention of complications in stroke must be taken into account. Further studies are needed to determine how HT occurs in ischemic stroke patients with ESRD as it causes a significant burden to the patient and family. 
